# Hydrogel-Shielded Ellagic Acid Nanoparticles Prolong Colonic Retention and Mitigate DSS-Induced Colitis via Reactive Oxygen Species Scavenging

**DOI:** 10.3390/foods14152559

**Published:** 2025-07-22

**Authors:** Ximei Ye, Tao Chen, Lihang Chen, Di Wu, Yinan Du, Jiangning Hu

**Affiliations:** SKL of Marine Food Processing & Safety Control, National Engineering Research Center of Seafood, Collaborative Innovation Center of Seafood Deep Processing, School of Food Science and Technology, Dalian Polytechnic University, Dalian 116034, China; 18770910433@163.com (X.Y.); 18305564864@163.com (T.C.); lhchen@xy.dlpu.edu.cn (L.C.); m13039998695@163.com (D.W.); dyn7381@163.com (Y.D.)

**Keywords:** reactive oxygen species, ellagic acid, nanoparticles, inflammatory bowel disease, colon

## Abstract

Inflammatory bowel disease (IBD) is characterized by oxidative stress imbalance and intestinal barrier disruption. Reducing excessive ROS has become a promising therapeutic strategy. Compared with conventional polyphenols, nanomaterials offer greater stability and bioavailability for ROS scavenging. Here, ellagic acid (EA) was converted into uniform nanoparticles (EAs) with reactive oxygen scavenging capacity through horseradish peroxidase (HRP)-mediated oxidative polymerization and subsequently encapsulated in the anti-gastric acid hydrogel F-DP to obtain the hybrid system F-DP@EAs. EAs reduced ROS, MDA, NO, IL-1β, and TNF-α levels in vitro, while increasing IL-4 and IL-10 expression, thus alleviating inflammation. Herein, F-DP@EAs prolonged intestinal retention time and exerted superior protective effects in the DSS-induced colitis model. Oral F-DP@EAs lowered DAI, preserved colon length, increased glutathione (GSH) and superoxide dismutase (SOD), decreased NO and MDA, restored zonula occludens-1 (ZO-1), and reduced mucosal lesions. These findings demonstrate that combining nanoparticle and hydrogel technologies markedly enhances the preventive and protective efficacy of EA, highlighting F-DP@EAs as a promising candidate for future IBD therapy.

## 1. Introduction

Inflammatory bowel disease (IBD) is a highly prevalent disorder characterized by persistent inflammation of colonic mucosa, disruption of the intestinal barrier, and compromised immune function [1]. While the pathogenesis of IBD is complex, evidence increasingly links it to oxidative damage caused by overabundant reactive oxygen species (ROS) production in gastrointestinal mucosa [2]. Current clinical regimens rely on small-molecule immunosuppressants such as the TNF-α inhibitor infliximab, anti-inflammatory agents including aminosalicylic acid (5-ASA), natural polyphenols, and kinase inhibitors targeting Janus kinase (JAKs) [3,4]. Prolonged administration of these drugs often leads to drug resistance, complications, and autoimmune disorders. ROS such as superoxide anions (•O_2_^−^), hydroxyl radicals (•OH), singlet oxygen (^1^O_2_), and hydrogen peroxide (H_2_O_2_) up-regulate pro-inflammatory cytokines, especially TNF-α, compromise mucosal integrity, induce DNA damage and apoptosis, increase intestinal permeability, aggravate mucosal injury, and facilitate pathogen invasion [5,6,7]. Consequently, limiting ROS accumulation is considered a promising preventive strategy for IBD.

Natural polyphenols, like curcumin, resveratrol, bilirubin, ellagic acid (EA), and others, are attractive candidates for IBD management because of their affordability, perceived safety, and combined antioxidant and anti-inflammatory properties [8,9,10]. However, low gastrointestinal stability, poor water solubility, rapid metabolism, and prompt elimination severely restrict the practical application of these compounds after oral administration [11,12]. EA, a ubiquitous constituent of various fruits, nuts, and plant tissues, exhibits potent antioxidant, anti-carcinogenic, and anti-human immunodeficiency virus activities [13,14,15]. Unfortunately, the oral bioavailability of EA remains alarmingly low owing to its limited solubility [16].

Nanotechnology offers new opportunities for IBD therapy [17,18]. For example, Xu et al. introduced dual-responsive nanoparticles (Rh2/LA-UASP NPs) composed of polymer LA-UASP grafted with asparagine polysaccharide, urocanic acid, and α-lipoic acid; these nanoparticles specifically delivered ginsenoside Rh2 to colitis sites, significantly alleviating ulcerative colitis and restoring intestinal microbial balance [4]. Compared with monomeric polyphenols, nano-systems generated through self-assembly of natural polyphenols provide superior ROS-scavenging capacity and, owing to their micro-scale dimensions, limit systemic absorption and reduce adverse reactions [19]. In addition, the negatively charged zeta potential and adhesive catechol groups enable such nano-systems to accumulate at inflamed intestinal sites, thereby prolonging local residence time [1].

Based on these findings, ellagic acid nanoparticles (EAS) with active oxygen scavenging capacity were successfully prepared by horseradish peroxidase-mediated enzymatic oxidation and then encapsulated in the anti-gastric acid F-DP (dopamine-modified folate hydrogel) hydrogel, previously reported in our laboratory for in vivo animal experiments [20]. The resulting gel–nanoparticle hybrid system (F-DP@EAs) significantly alleviated dextran sodium sulfate (DSS)-induced colitis in mice. In vitro, free EAs markedly decreased oxidative stress-derived ROS and mitigated cellular inflammation, whereas encapsulation within F-DP extended intestinal retention and enhanced therapeutic efficacy in vivo. These results demonstrate that the integration of nanoparticle and hydrogel technologies constitutes a promising precautionary approach for IBD.

## 2. Materials and Methods

### 2.1. Materials

Ellagic acid (EA, purity ≥ 98%) was sourced from Macklin (Shanghai, China), DSS (dextran sulfate sodium) was sourced from Yeasen Biotechnology Co., Ltd. (Shanghai, China), and 5-aminosalicylic acid (5-ASA) was purchased from Shanghai McLean Biochemical Technology Co., Ltd. (Shanghai, China). ELISA kits detecting TNF-α, IL-1β, IL-4, and IL-10 were obtained from Shanghai Enzyme-linked Biotechnology Co., Ltd (Shanghai, China). RAW 264.7 cells were obtained from Wuhan Procell Life Science & Technology Co., Ltd (Wuhan, China).

### 2.2. Preparation of EA Nanoparticles

EA (100 mg) was completely dissolved in dimethyl sulfoxide (DMSO) at room temperature under magnetic stirring. PEG 4000 (100 mg) and horseradish peroxidase (HRP, 5 mg, 300 U) were subsequently added until fully dissolved. Hydrogen peroxide (H_2_O_2_, 0.3%, 600 μL) was then introduced dropwise, causing the solution to darken immediately. The reaction mixture was stirred for 12 h. The resulting suspension was dialyzed against deionized water for 24 h (MWCO = 3.5 kDa), centrifuged at 10,000 rpm to collect the precipitate, and freeze-dried. For Fourier-transform infrared (FT-IR) spectroscopy, the dried powder was pressed into KBr pellets and analyzed on an FT-IR spectrometer (PerkinElmer, Waltham, MA, USA). Intermolecular interactions were further confirmed by X-ray diffraction (XRD, Shimadzu XRD-7000s) and UV–visible spectroscopy (UV-2600, Shimadzu Corporation, Kyoto, Japan).

### 2.3. Assembly and Analysis of F-DP@EAs Nanoparticle Hydrogel

To prolong intestinal residence, EAs were encapsulated in the previously reported anti-gastric acid hydrogel F-DP. Briefly, EAs were uniformly dispersed in a solution of ferulic acid modified with dopamine hydrochloride (FA-DA) and subsequently mixed with a protocatechuic acid (PCA) solution. The mixture was left undisturbed until a hydrogel formed. Rheological properties were evaluated at predefined intervals using hybrid rheometer (HR-2, TA Instruments, New Castle, DE, USA) equipped with 25 mm parallel-plate geometry.

### 2.4. Cytotoxicity Assay

RAW 264.7 cells were placed in 5% CO_2_ incubator and cultured in complete medium containing 10% fetal bovine serum and 1% penicillin streptomycin. Cells were plated in 96-well plates at a density of 1 × 10^4^ cells per well and incubated for 24 h to allow for adherence. Subsequently, a series of concentrations of ellagic acid nanoparticles (EAs) was applied for an additional 24 h. Cell viability was assessed with a commercial live/dead staining kit, and fluorescence images were acquired using an Nikon Ti–S inverted fluorescence microscope (Nikon, Tokyo, Japan).

### 2.5. Anti-Inflammatory Activity Assessed In Vitro

To evaluate anti-inflammatory properties, RAW 264.7 cells were cultured in 6-well plates at a density of 1 × 10^5^ cells per well and incubated for 24 h. Subsequently, cells were then exposed to free EA or EAs for 24 h, followed by lipopolysaccharide (LPS) stimulation for 24 h and hydrogen peroxide (H_2_O_2_) challenge for 4 h. SOD activity, MDA content, and NO levels were quantified with corresponding assay kits, whereas interleukin-1 β (IL-1 β), tumor necrosis factor-α (TNF-α), interleukin-10 (IL-10), and interleukin-4 (IL-4) were determined via enzyme-linked immunosorbent assay.

### 2.6. Intracellular ROS Assay

RAW 264.7 cells were seeded in 24-well plates at a density of 50,000 cells per well and cultured for 24 h. They were then treated with free EA or EAs for another 24, followed by induction with LPS for 12 h and H_2_O_2_ for 4 h. Subsequently, cells were incubated with DCFH-DA for 20 min as per manufacturer’s instructions and washed three times with PBS to remove excess DCFH-DA and reduce background interference. Finally, cellular images were captured using a fluorescence microscope (Nikon Ti–S, Nikon Corporation).

### 2.7. Mitochondrial Membrane Potential (MMP) Assay

RAW 264.7 cells were seeded in 24-well plates at a density of 50,000 cells per well and cultured for 24 h. Subsequently, cells were treated with free EA or EAs for 24 h. Cells were then stimulated with LPS for 12 h and H_2_O_2_ for 4 h, followed by staining with JC-1 for 3 h. Fluorescence images reflecting MMP changes were captured with a fluorescence microscope (Nikon Ti–S, Nikon Corporation).

### 2.8. In Vivo Intestinal Retention

Intestinal retention of nanoparticles was assessed in mice using fluorescein isothiocyanate (FITC) as a tracer. Mice were divided into 4 groups with the following specific groupings and treatments: healthy mice intragastrically administered F-DP@EAs, healthy mice intragastrically administered EAs, DSS-induced IBD mice intragastrically administered F-DP@EAs, and DSS-induced IBD mice intragastrically administered EAs. The intragastric administration volume for each mouse was adjusted to 300 μL. At predetermined time points (2, 4, 8, 12, 24, and 48 h) following intragastric administration, mice were anesthetized, euthanized, and dissected. Major organs (heart, liver, spleen, kidneys, lungs, stomach, and intestines) were rapidly excised and visualized with a versatile in vivo imaging system (MIIS XFP-BIX, Ami HTX, Spectral Instrument Imaging, Tucson, AZ, USA).

### 2.9. Biosafety of EAs

The cytotoxicity of EAs toward RAW 264.7 macrophages was evaluated with MTT assay. RAW 264.7 cells were grown in 96-well plates at a density of 10,000 cells per well and left to incubate for 12 h. Serial concentrations of EAs were then applied for 24 h at 37 °C. Following this, 15 μL of MTT solution was added, and plates were continue incubated for 4 h. The resulting formazan crystals were dissolved in dimethyl sulfoxide (DMSO), and absorbance was measured at 490 nm. Hemolytic activity was evaluated with mouse red blood cells. Whole blood in anticoagulant tubes was centrifuged at 1500 rpm for 10 min, and pellets were resuspended in saline to create a 2% (*v*/*v*) suspension. Aliquots (1 mL) were treated with phosphate-buffered saline (PBS), deionized water, free EA, or EAs and incubated for 1 h. Post-centrifugation, supernatants were collected and analyzed at 570 nm. The hemolysis rate was calculated as described (Figure S1A).

### 2.10. Preventive Effects of EAs in DSS-Induced Colitis

Animal Experiment Ethics Committee of Dalian Polytechnic University approved all procedures involving animals, with the approval number PLPU2023101. Under standard conditions (20–26 °C, 40–70% relative humidity, 12/12 h light/dark cycle), Balb/c mice were acclimated for 7 days with free access to water and food. Animals were randomly allocated to seven groups (n = 6): (1) Control; (2) DSS; (3) EA (75 mg kg^−1^ bw); (4) EAs (75 mg kg^−1^ bw); (5) F-DP@EAs (EAs 75 mg kg^−1^ bw); (6) Positive group (5-aminosalicylate, 100 mg kg^−1^ bw); and (7) F-DP (0.2 mL per mouse). Each formulation (0.2 mL) was administered via oral gavage once daily in the afternoon for 21 d. On day 22, drinking water of all groups except the Control group was replaced with 3 % (*w*/*v*) DSS solution. Oral dosing continued until day 28. Disease activity index (DAI) was calculated daily from body weight change, stool consistency, occult blood, and gross bleeding, following the published scoring system [21]. At the endpoint, mice were euthanized, colons were excised and measured, and tissues were stored at −80 °C for later analyses.

### 2.11. Histological Examination of Colon

Colon tissues were fixed in paraformaldehyde and embedded in paraffin. Paraffin-embedded blocks were then sectioned into 5 μm thick slices, which were subsequently transferred to xylene and graded ethanol for processing. Following this, sections were stained with hematoxylin–eosin (H&E) and Masson’s trichrome stain. Finally, histological morphology of the colon was observed under a bright-field microscope, and images were captured.

### 2.12. Cytokine and Oxidative Stress Analysis in Colonic Tissue

Colon tissues were mixed with PBS (pH 6.0) at a weight-to-volume ratio of 1:9 and homogenized using an IKA homogenizer(IKA Industrial Equipment Group, Staufen im Breisgau, Germany). They were then centrifuged at 10,000 rpm for 10 min at 4 °C to collect supernatant. Total protein concentration in the supernatant was quantified using a BCA assay kit. IL-1β, IL-4, IL-10, and TNF-α concentrations were measured using commercial ELISA kits, while MDA and SOD levels were evaluated with assay kits from Beijing Solarbio Science & Technology Co., Ltd., Beijing, China.

### 2.13. Statistical Analysis

Data are presented as mean ± standard deviation. Three independent experiments were performed, and at least three replicates were analyzed in each experiment. Fluorescence intensity was quantified with ImageJ (ImageJ 1.52a). Statistical comparisons were performed using SPSS 20.0 software, with one-way analysis of variance (ANOVA) applied for analysis. Graphs were created using GraphPad Prism 8.0.

## 3. Results and Discussion

### 3.1. Fabrication and Characterization of EAs and F-DP@EAs

**Figure 1:**
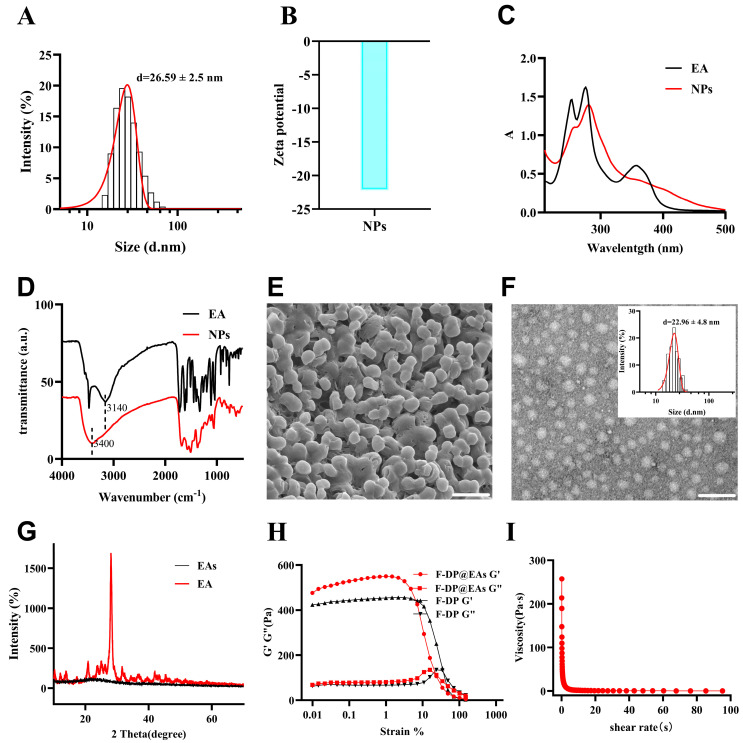
Preparation and characterization of EAs: (**A**) particle size distribution and (**B**) ζ-potential of EAs; (**C**) ultraviolet absorption spectra comparison between EA and EAs; (**D**) FT-IR spectral analysis of EA and EAs; (**E**) SEM and TEM images (**F**) of EAs; (**G**) XRD spectra of EA and EAs; (**H**) oscillatory strain sweeps of F-PP hydrogels and F-DP@EAs; (**I**) rheological flow curves of F-DP@EAs measured at shear rates between 0.1 and 100 s^−1^ at 25 °C. Scale bar = 100 nm.

EAs were produced via horseradish-peroxidase catalyzed oxidative polymerization. At room temperature, spherical particles with narrow size distribution (PDI < 0.1) were obtained, as shown in Figure 1A,E. Particle size was regulated by adjusting the amounts of PEG and HRP, and the resulting distribution was validated by employing dynamic light scattering (DLS) and TEM. (Table S1). Zeta-potential analysis revealed a negative surface charge, indicating good colloidal stability in an aqueous medium (Figure 1B). UV–Vis and FT-IR spectroscopy were employed to elucidate structural changes after polymerization. In the UV–Vis spectra (Figure 1C), the main absorption band decreased in intensity and red-shifted, implying π–π stacking of aromatic rings in EAs [22]. Correspondingly, the C–OH stretching vibration shifted from 3140 cm^−1^ in EA to 3400 cm^−1^ in EAs, consistent with intramolecular hydrogen bond formation (Figure 1D) [23]. A distinct peak at 1512 cm^−1^ further supported π–π interactions within the polymerized network [24]. X-ray diffraction (XRD) patterns exhibited reduced crystallinity (Figure 1G), corroborating these spectroscopic findings. TEM imaging gave an average particle diameter of 22.96 ± 4.8 nm, slightly smaller than the 26.59 ± 2.5 nm determined by DLS, a difference ascribed to particle swelling in solution (Figure 1F) [4]. The uniform dispersion and nanoscale size demonstrate that the fabricated EAs effectively overcome the poor solubility and limited bioavailability of free EA. For enhanced gastrointestinal transit and prolonged intestinal residence, EAs were encapsulated within the F-DP hydrogel. Encapsulation did not alter the hydrogel’s rheological properties; the material still possessed favorable mechanical properties and shear thinning characteristics, indicating that it retained excellent injectability and remained suitable for oral administration (Figure 1H,I).

### 3.2. Cellular Uptake Behavior of EAs by Macrophages

**Figure 2:**
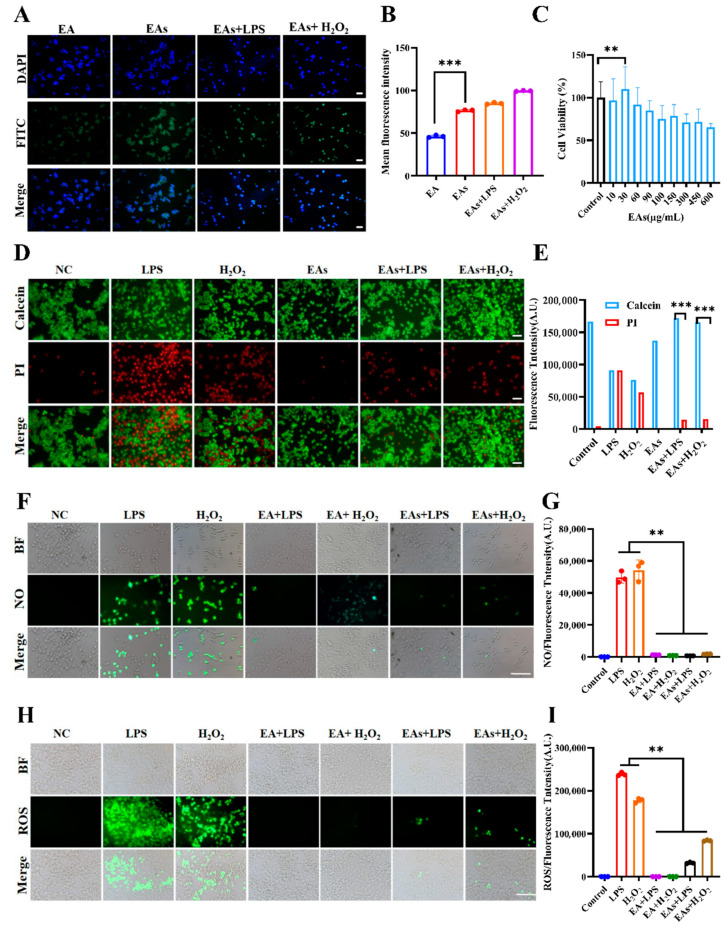
The study examines the reactive oxygen species scavenging effects of EAs through various analyses: (**A**) fluorescence images and (**B**) mean fluorescence intensity to assess cellular uptake in cells treated with EA and EAs; (**C**) cell viability analysis; (**D**) fluorescence images and (**E**) average fluorescence intensity for live and dead staining of macrophages treated with EA and EAs; (**F**) fluorescence imaging and (**G**) average fluorescence intensity for intracellular NO; (**H**) fluorescence imaging and (**I**) average fluorescence intensity for intracellular ROS in cells treated with EA and EAs. Scale bar = 200 µm. ** *p* < 0.01, *** *p* < 0.001.

Maximizing the bioavailability and anti-inflammatory effects of nanoparticles relies on efficient intracellular uptake. The internalization of EAs by RAW 264.7 macrophages was therefore examined (Figure 2A,B). Relative to free EA, the fluorescence intensity in the EAs treated group cells was significantly enhanced, indicating a significant increase in cell uptake of EAs. This enhancement can be attributed to two factors: first, the improved water solubility and stability of EA following nanoparticle preparation, which facilitates intracellular accumulation; second, its nanoscale size. According to reports, nanoscale materials are readily internalized by intestinal epithelial cells, neutrophils, macrophages, and other immune cells via endocytosis, which is likely the reason for the increased cellular uptake of EAs [25]. Notably, cells stimulated with lipopolysaccharide (LPS) and hydrogen peroxide (H_2_O_2_) displayed stronger green fluorescence, reflecting higher nanoparticle uptake. This increase was probably related to LPS- and H_2_O_2_-induced elevations in membrane permeability, which facilitated greater entry of EAs and potentially amplified their anti-inflammatory efficacy [26].

### 3.3. ROS-Scavenging Effects of EAs in Macrophages

**Figure 3:**
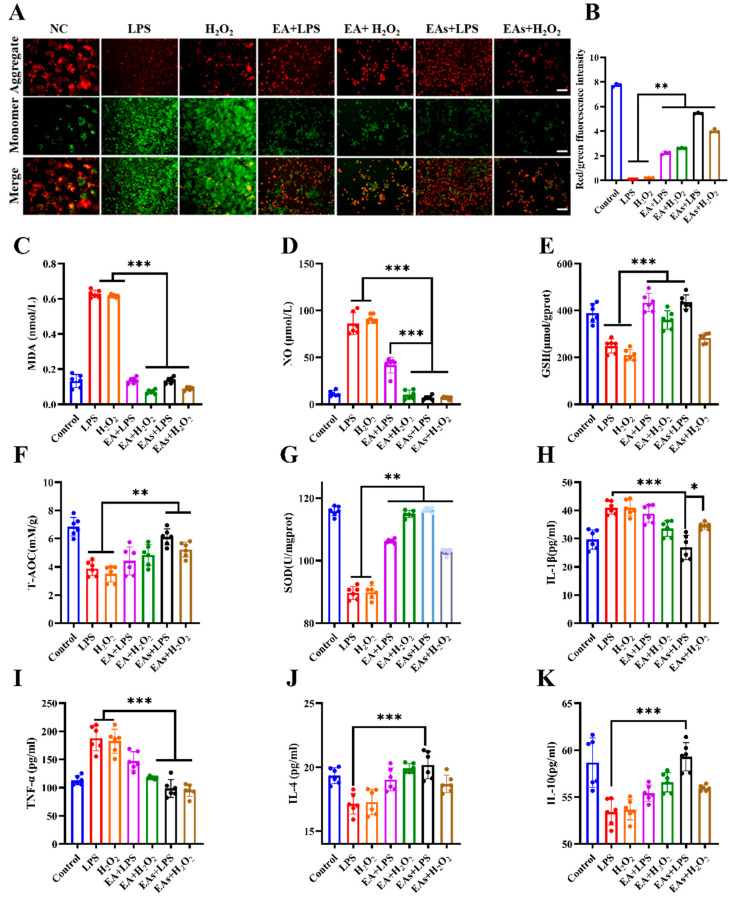
Anti-inflammatory effects of EAs in macrophages: (**A**) JC-1 fluorescence images of RAW 264.7 cells treated with EA or EAs; (**B**) corresponding mean fluorescence intensity; expression levels of oxidative stress markers MDA (**C**), NO (**D**), and GSH (**E**), total antioxidant capacity (T-AOC, (**F**)), SOD (**G**), and cytokines IL-1β (**H**), TNF-α (**I**), IL-4 (**J**), and IL-10 (**K**) with different treatments. Scale bar = 200 µm. Data are presented as mean ± SD (n = 6). * *p* < 0.05; ** *p* < 0.01; *** *p*<0.001.

The capacity of EAs to scavenge intracellular reactive oxygen species (ROS) was assessed to corroborate their anti-inflammatory potential. After pretreatment with free EA or EAs, cells were challenged with LPS and H_2_O_2_ to induce oxidative stress (Figure 2D,E). Intense red fluorescence in the LPS- and H_2_O_2_-treated groups confirmed substantial ROS production and ensuing apoptosis. Both EA and EAs markedly attenuated the red signal, demonstrating pronounced cytoprotective activity. Fluorescence in EAs-only cells resembled that of the untreated control, indicating negligible cytotoxicity, which is consistent with the results of effects of EA and EAs on cell viability in Figure 2C. Nitric oxide (NO) levels were likewise monitored (Figure 2F,G). LPS and H_2_O_2_ elicited strong green fluorescence, whereas treatment with EA or EAs sharply reduced the signal, signifying potent NO scavenging capability. Consistent outcomes were observed when overall ROS levels were quantified (Figure 2H,I). This suggests that EAs possess excellent antioxidant properties, enabling them to scavenge various reactive oxygen species generated in cells and thereby alleviate cellular inflammation. To verify protection against acute oxidative injury, the JC-1 probe was used to evaluate mitochondrial membrane potential according to the previously reported method (Figure 3A,B) [27]. LPS or H_2_O_2_ exposure caused a shift from red to green fluorescence, demonstrating the loss of transmembrane potential and early-phase apoptosis. In contrast, cells pre-incubated with EA or EAs exhibited minimal green fluorescence, indicating preservation of mitochondrial function. The green fluorescence attenuation effect of cells treated with EAs in the LPS model was better than that of all other drug treatment groups. This suggests that the protective effect was especially pronounced for EAs in the LPS model, underscoring their superior ability to counteract acute oxidative damage.

### 3.4. Anti-Inflammatory Effects of EAs in Macrophages

The in vitro anti-inflammatory activity of EAs was examined in RAW 264.7 macrophages. Cells were pre-incubated with free EA or EAs and subsequently challenged with LPS or H_2_O_2_. Pro- and anti-inflammatory mediators were then quantified (Figure 3C–K). Both treatments markedly lowered levels of the pro-inflammatory markers NO, MDA, TNF-α, and IL-1β while increasing anti-inflammatory cytokines IL-10 and IL-4, demonstrating significant anti-inflammatory activity. In the LPS model, EAs produced a greater reduction in pro-inflammatory mediators than free EA, including NO, IL-1β, and TNF-α, an effect probably linked to higher cellular uptake of nanoparticles. In contrast, free EA showed stronger protection in the H_2_O_2_ model, likely because the molecular form more effectively scavenged H_2_O_2_-derived ROS. Pretreatment with either formulation also enhanced cellular antioxidant capacity. Intracellular glutathione (GSH) and superoxide dismutase (SOD) activities increased, with the elevation being more pronounced in LPS-stimulated cells, indicating that EAs may have a relieving effect on IBD. These results indicate that the nanoscale formulation confers superior uptake and bioavailability, allowing for more effective interaction with intracellular targets and stronger antioxidant and anti-inflammatory effects.

### 3.5. Biodistribution of EAs and F-DP@EAs In Vivo

**Figure 4:**
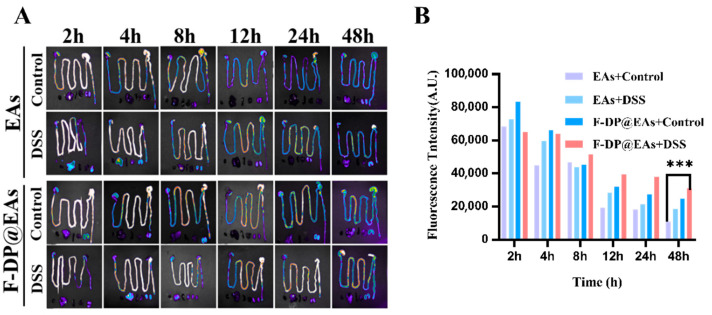
Biodistribution of EAs and F-DP@EAs in vivo: (**A**) fluorescence imaging of gastrointestinal tract in DSS-induced IBD mice and healthy mice after oral administration of free EAs or F-DP@EAs; (**B**) corresponding mean fluorescence intensity. *** *p*<0.001.

Efficient traversal of phenolic nanoparticles through the gastrointestinal tract and their subsequent accumulation in inflamed colonic tissue are both crucial for alleviating IBD [28,29]. To meet this challenge, EAs were encapsulated within the anti-gastric acid hydrogel F-DP that had been developed previously. This strategy was designed to protect EAs from the harsh gastrointestinal milieu and to extend their colonic residence. As illustrated in Figure 4A,B, the colons of DSS-induced IBD mice exhibited enhanced fluorescence intensity, likely due to inflammation-induced increases in intestinal permeability [30]. In healthy mice, fluorescence from free EAs disappeared within 12 h, suggesting premature degradation of the nanoparticles in the absence of hydrogel protection [31]. F-DP@EAs maintained strong fluorescence 48 h post-administration, indicating that hydrogel encapsulation protected EAs from enzymatic and pH-dependent degradation, enhancing their retention in the colon and improving preventive efficacy.

### 3.6. Preventive Effects of EAs in DSS-Induced IBD Mice

**Figure 5:**
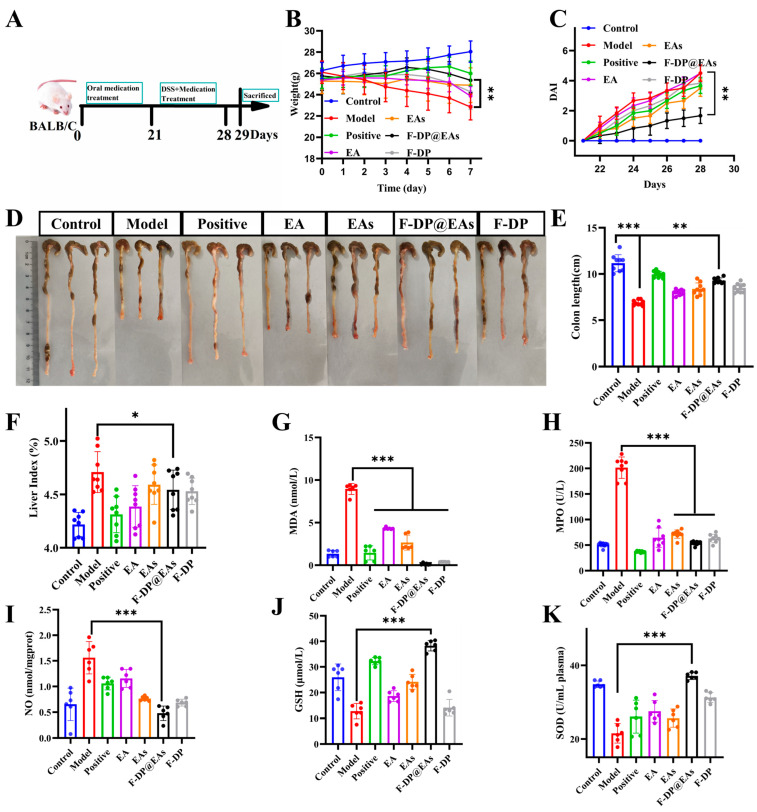
Therapeutic effects of EA, EAs, and F-DP@EAs in DSS-induced colitis mice: (**A**) experimental timeline; (**B**) daily body weight change; (**C**) DAI scores after 3% DSS administration; (**D**) representative photographs of excised colons; (**E**) colon length; (**F**) liver index; serum levels of MDA (**G**), MPO (**H**), NO (**I**), GSH (**J**), and SOD (**K**). Data are expressed as mean ± SD (n = 6); * *p* < 0.05; ** *p* < 0.01; *** *p* < 0.001.

The in vivo efficacy of EAs was assessed in a dextran sodium sulfate (DSS) colitis model. The experimental design is illustrated in Figure 5A. From the experimental results, it can be seen that mice in the DSS group exhibited rapid body weight loss and elevated disease activity index (DAI) scores, whereas F-DP@EAs markedly mitigated weight loss and reduced DAI relative to the DSS group (Figure 5B,C). Oral administration of EA, EAs, or F-DP@EAs also prevented DSS-induced colon shortening, with the greatest improvement observed in the F-DP@EAs cohort (Figure 5D,E). Because colitis is often accompanied by hepatomegaly and an increased liver index [32], the significantly lower liver index in the F-DP@EAs group confirmed its superior anti-inflammatory efficacy (Figure 5F). Serum analysis showed that EA, EAs, and F-DP@EAs decreased malondialdehyde (MDA), nitric oxide (NO), and myeloperoxidase (MPO) activities compared with DSS alone, indicating effective suppression of systemic inflammation. Concomitantly, glutathione (GSH) and superoxide dismutase (SOD) activities were elevated, reflecting enhanced antioxidant defenses (Figure 5G–K). Consistent with in vitro findings, these results indicate that in the DSS-induced IBD model, EAs exhibit a strong capacity to scavenge reactive oxygen species generated in mice. Moreover, compared with the group treated with EAs alone, the F-DP@EAs group produced the most pronounced reductions in inflammatory markers and the greatest improvements in oxidative stress indices. This suggests that encapsulating EAs in the hydrogel enhances its accumulation in the colon, thereby strengthening its preventive effect on IBD.

**Figure 6:**
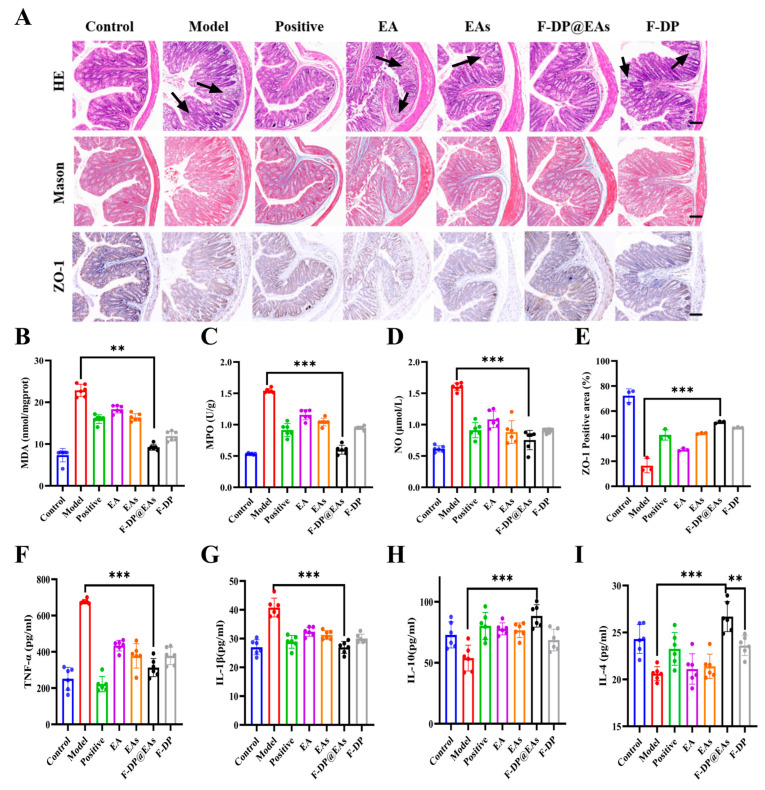
Therapeutic effects of EA, EAs, and F-DP@EAs in mice with DSS-induced colitis: (**A**) H&E staining, Masson’s trichrome staining(the arrow indicates the region of damage), and ZO-1 immunohistochemistry of colonic sections (scale bar = 200 μm); (**B**) MDA, (**C**) MPO, and (**D**) NO levels in colon tissue; (**E**) quantitative analysis of ZO-1 staining; (**F**) TNF-α, (**G**) IL-1β, (**H**) IL-10, and (**I**) IL-4 levels. Data are expressed as mean ± SD (n = 6). ** *p* < 0.01; *** *p*<0.001.

Histological alterations were evaluated by haematoxylin–eosin (H&E) and Masson’s trichrome staining. As illustrated in Figure 6A, colonic tissue from the DSS group exhibited pronounced inflammatory-cell infiltration, severe goblet-cell depletion, and crypt loss. Treatment with EA, EAs, or F-DP@EAs markedly alleviated inflammation and restored goblet cells and crypt architecture. The F-DP@EAs group, due to the protective effect of hydrogel on EAs, showed significantly better effects than the single EAs treatment group, and the morphology was similar to that of control group. Masson’s staining further confirmed mucosal recovery. Extensive loss of collagen fibres was evident in the DSS group, whereas blue-stained collagen deposits reappeared after all treatments, indicating protection of the epithelial barrier. To assess tight-junction integrity, expression of zonula occludens-1 (ZO-1) was quantified [33]. ZO-1 staining increased significantly in the EA, EAs, and F-DP@EAs groups, and the positive area was largest in the F-DP@EAs cohort (Figure 6E), demonstrating superior preservation of the mucosal barrier. The primary reason is that hydrogels enhance the accumulation of EAs in the colon, thereby alleviating inflammation and mitigating intestinal barrier damage induced by IBD. Collectively, results confirm that oral F-DP@EAs effectively prevented DSS-induced IBD.

Inflammatory mediators in colonic tissue were quantified to corroborate anti-inflammatory efficacy of F-DP@EAs. In the F-DP@EAs group, MDA, MPO, and NO levels were significantly lower than those in the DSS group (Figure 6B–D), indicating reduced mucosal inflammation. There was a notable down-regulation of pro-inflammatory cytokines TNF-α and IL-1β, occurring alongside an up-regulation of anti-inflammatory cytokines IL-4 and IL-10 in the F-DP@EAs group (Figure 6F–I). The results are consistent with the initially envisioned effects of this study, confirming that oral F-DP@EAs effectively enhance the accumulation of EAs in the colon, mitigate colonic inflammation and prevent DSS-induced IBD.

### 3.7. In Vivo Safety Assessment

**Figure 7:**
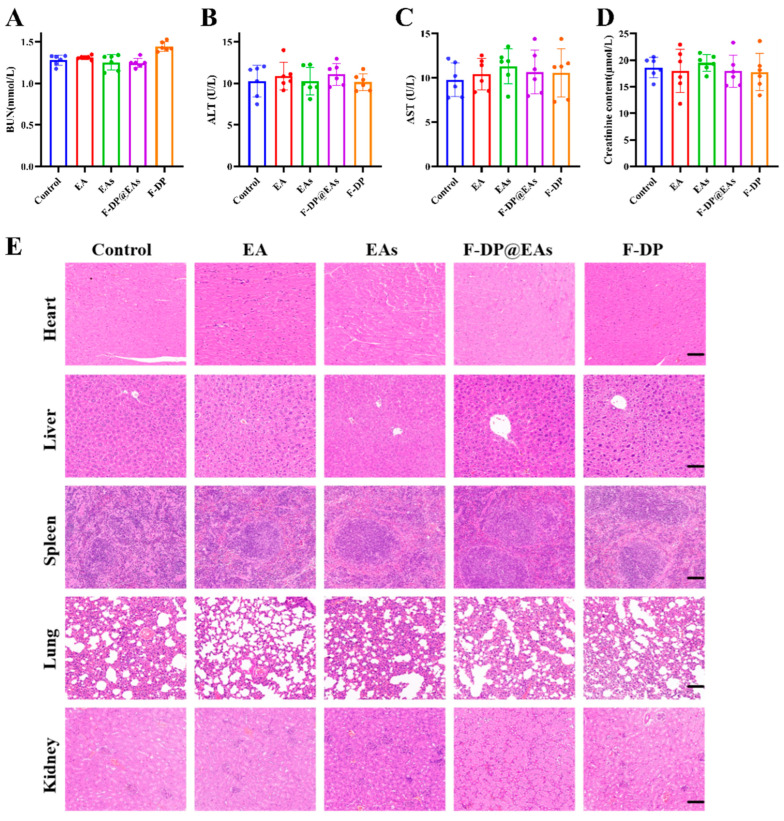
In vivo safety assessment: (**A**) BUN (blood urea nitrogen); (**B**) ALT (alanine aminotransferase); (**C**) AST (aspartate aminotransferase); (**D**) creatinine; (**E**) representative hematoxylin–eosin (H&E) of sections of the heart, liver, spleen, lung, and kidney. Scale bar: 100 μm; values are mean ± SD (n = 6).

Biosafety was evaluated by monitoring liver and kidney function after oral administration of EA, EAs, F-DP@EAs, or F-DP. As anticipated, administration of EA, EAs, F-DP@EAs, or F-DP in mice showed no significant changes in liver and kidney function indicators (ALT, AST, CR, and BUN) compared to control group, indicating an absence of hepatotoxicity or nephrotoxicity (Figure 7A–D). Consistently, as shown in Figure 7E, haematoxylin–eosin-stained sections of the heart, liver, spleen, lung, and kidney displayed intact histoarchitecture without inflammatory infiltration or tissue damage, indicating that F-DP@EAs No toxic side effects on organs in mice [34]. These research findings confirm that F-DP@EAs exhibit no obvious toxic side effects and possess excellent in vivo safety, thereby providing robust support for their translation from laboratory research to clinical applications, with broad prospects for use.

## 4. Conclusions

Ellagic acid nanoparticles (EAs) were synthesized by enzymatic oxidative polymerization and displayed strong reactive oxygen species scavenging activity, favorable biosafety, and efficient cellular uptake. In vitro, EAs effectively eliminated intracellular ROS and NO and reduced pro-inflammatory, including TNF-α and IL-1β, thereby suppressing LPS- and H_2_O_2_-induced inflammatory responses. Encapsulation within the anti-gastric acid hydrogel F-DP enabled EAs to withstand the gastrointestinal environment and markedly prolonged their colonic residence. F-DP@EAs significantly decreased the disease activity index, prevented colon shortening, and improved antioxidant defenses by increasing GSH and SOD and reducing MDA and NO in a DSS-induced colitis model. Histological analyses confirmed restoration of the mucosal barrier, recovery of ZO-1 expression, and attenuation of inflammatory lesions. These findings emphasize the significant potential of EAs as ROS-scavenging agents in preventing inflammation and managing inflammatory pathological processes, with broad application prospects.

## Data Availability

The original contributions presented in the study are included in the article/Supplementary Material, further inquiries can be directed to the corresponding author.

## Supplementary Materials

The following supporting information can be downloaded at https://www.mdpi.com/article/10.3390/foods14152559/s1, Figure S1: (A) Statistical analysis of the hemolysis rate (n = 5). (B) Image of the result of the hemolysis test; Table S1: Statistical parameters of prepared EAs.

## References

[1] Sun W., Chen Y., Wang L., Wang Z., Liu S., Zhang M., Liu Y., Li Q., Zhang H. (2023). Gram-scale preparation of quercetin supramolecular nanoribbons for intestinal inflammatory diseases by oral administration. Biomaterials.

[2] Tan C., Fan H., Ding J., Han C., Guan Y., Zhu F., Wu H., Liu Y., Zhang W., Hou X. (2022). ROS-responsive nanoparticles for oral delivery of luteolin and targeted therapy of ulcerative colitis by regulating pathological microenvironment. Mater. Today Bio.

[3] Cai W.-Q., Liang W., Li D., Dai W., Li Z., Wei X., Cheng L., Zhang B.-B., Yang Q. (2025). Reactive Oxygen Species-Responsive Polymer Drug Delivery System Targeted Oxidative Stressed Colon Cells to Ameliorate Colitis. ACS Nano.

[4] Xu Y., Zhu B.-W., Sun R., Li X., Wu D., Hu J.-N. (2023). Colon-Targeting Angelica sinensis Polysaccharide Nanoparticles with Dual Responsiveness for Alleviation of Ulcerative Colitis. ACS Appl. Mater. Interfaces.

[5] Liu Y., Cheng Y., Zhang H., Zhou M., Yu Y., Lin S., Jiang B., Zhao X., Miao L., Wei C.-W. (2020). Integrated cascade nanozyme catalyzes in vivo ROS scavenging for anti-inflammatory therapy. Sci. Adv..

[6] Ge J., Jia B., Wang Y., Ma Y., Sun X., Dong J., Jiang S., Li Z. (2024). DNA Nanostructures Treat Inflammatory Bowel Disease through ROS Scavenging and Gut Microbiota Modulation. Adv. Funct. Mater..

[7] Zhao N., Han Y.-J., Wang C., Li J., Song L.-H., Lv L.-P., Ma P., Deng J., Zhang Y.-Y. (2025). Two Birds with One Stone: Empowering Probiotic with Nanoenzyme for the Treatment of Inflammatory and Anemia through Oral Administration. ACS Appl. Mater. Interfaces.

[8] Zhang J., Xie H., Wang T., Zhang H., Yang Z., Yang P., Li Y., Ma X., Gu Z. (2022). Epicatechin-assembled nanoparticles against renal ischemia/reperfusion injury. J. Mater. Chem. B.

[9] Wang T., Fan Q., Hong J., Chen Z., Zhou X., Zhang J., Dai Y., Jiang H., Gu Z., Cheng Y. (2021). Therapeutic Nanoparticles from Grape Seed for Modulating Oxidative Stress. Small.

[10] Hu Q., Li J., Wang T., Xu X., Duan Y., Jin Y. (2024). Polyphenolic Nanoparticle-Modified Probiotics for Microenvironment Remodeling and Targeted Therapy of Inflammatory Bowel Disease. ACS Nano.

[11] Wang T., Zhang J., Zhang H., Bai W., Dong J., Yang Z., Yang P., Gu Z., Li Y., Chen X. (2022). Antioxidative myricetin-enriched nanoparticles towards acute liver injury. J. Mater. Chem. B.

[12] Miao R., Jin F., Wang Z., Lu W., Liu J., Li X., Zhang R.X. (2022). Oral delivery of decanoic acid conjugated plant protein shell incorporating hybrid nanosystem leverage intestinal absorption of polyphenols. Biomaterials.

[13] Jin H., Che S., Wu K., Wu M. (2022). Ellagic acid prevents gut damage via ameliorating microbe-associated intestinal lymphocyte imbalance. Food Funct..

[14] Duan J., Pan J., Sun M., Fang Y. (2022). Comparative multiomics study of the effects of Ellagic acid on the gut environment in young and adult mice. Food Res. Int..

[15] Han B., Shi L., Bao M.-Y., Yu F.-L., Zhang Y., Lu X.-Y., Wang Y., Li D.-X., Lin J.-C., Jia W. (2024). Dietary ellagic acid therapy for CNS autoimmunity: Targeting on Alloprevotella rava and propionate metabolism. Microbiome.

[16] Ha W., Ma R., Kang J.-Y., Iradukunda Y., Shi Y.-P. (2024). Green and shape-tunable synthesis of ellagic acid crystalline particles by tannic acid for neuroprotection against oxidative stress. Biomater. Sci..

[17] Zhang X., Yuan Z., Wu J., He Y., Lu G., Zhang D., Zhao Y., Wu R., Lv Y., Cai K. (2023). An Orally-Administered Nanotherapeutics with Carbon Monoxide Supplying for Inflammatory Bowel Disease Therapy by Scavenging Oxidative Stress and Restoring Gut Immune Homeostasis. ACS Nano.

[18] Luo R., Liu J., Cheng Q., Shionoya M., Gao C., Wang R. (2024). Oral microsphere formulation of M2 macrophage-mimetic Janus nanomotor for targeted therapy of ulcerative colitis. Sci. Adv..

[19] Rosso A., Andretto V., Chevalier Y., Kryza D., Sidi-Boumedine J., Grenha A., Guerreiro F., Gharsallaoui A., La Padula V., Montembault A. (2021). Nanocomposite sponges for enhancing intestinal residence time following oral administration. J. Control. Release.

[20] Ye X., Chen T., Du Y., Zhao R., Chen L., Wu D., Hu J. (2025). Folic acid-based hydrogels co-assembled with protocatechuic acid for enhanced treatment of inflammatory bowel disease. Colloids Surfaces B Biointerfaces.

[21] Wu Z., Huang S., Li T., Li N., Han D., Zhang B., Xu Z.Z., Zhang S., Pang J., Wang S. (2021). Gut microbiota from green tea polyphenol-dosed mice improves intestinal epithelial homeostasis and ameliorates experimental colitis. Microbiome.

[22] Yi Z., Chen G., Chen X., Ma X., Cui X., Sun Z., Su W., Li X. (2020). Preparation of Strong Antioxidative, Therapeutic Nanoparticles Based on Amino Acid-Induced Ultrafast Assembly of Tea Polyphenols. ACS Appl. Mater. Interfaces.

[23] Yuan C., Levin A., Chen W., Xing R., Zou Q., Herling T.W., Challa P.K., Knowles T.P.J., Yan X. (2019). Nucleation and Growth of Amino Acid and Peptide Supramolecular Polymers through Liquid–Liquid Phase Separation. Angew. Chem. Int. Ed. Engl..

[24] Wang H., Wang L., Guo S., Liu Z., Zhao L., Qiao R., Li C. (2022). Rutin-Loaded Stimuli-Responsive Hydrogel for Anti-Inflammation. ACS Appl. Mater. Interfaces.

[25] Li Q., Zeng M., Pu X., Tang Q., Yang Q., Zhang L. (2025). Melittin-Loaded Multifunctional Nanozyme for Ulcerative Colitis Treatment via Enzyme-Immunotherapy and Ferroptosis Inhibition. Adv. Funct. Mater..

[26] Park B., Han G., Jin D.Y., Gil K.C., Shin D., Lee J., Park J.Y., Jang H., Park D., Lee S. (2024). Mucoadhesive Mesalamine Prodrug Nanoassemblies to Target Intestinal Macrophages for the Treatment of Inflammatory Bowel Disease. ACS Nano.

[27] Hua Z., Zhang X., Zhao X., Zhu B.-W., Liu D., Tan M. (2023). Hepatic-targeted delivery of astaxanthin for enhanced scavenging free radical scavenge and preventing mitochondrial depolarization. Food Chem..

[28] Zhang X., Yang H., He Y., Zhang D., Lu G., Ren M., Lyu Y., Yuan Z., He S. (2025). Yeast-Inspired Orally-Administered Nanocomposite Scavenges Oxidative Stress and Restores Gut Immune Homeostasis for Inflammatory Bowel Disease Treatment. ACS Nano.

[29] Ye R., Guo J., Yang Z., Wang Z., Chen Y., Huang J., Dong Y. (2025). Somatostatin and Mannooligosaccharide Modified Selenium Nanoparticles with Dual-Targeting for Ulcerative Colitis Treatment. ACS Nano.

[30] Turner J.R. (2009). Intestinal mucosal barrier function in health and disease. Nat. Rev. Immunol..

[31] Zhao X., Wang L., Fu Y.-J., Yu F., Li K., Wang Y.-Q., Guo Y., Zhou S., Yang W. (2025). Inflammatory Microenvironment-Responsive Microsphere Vehicles Modulating Gut Microbiota and Intestinal Inflammation for Intestinal Stem Cell Niche Remodeling in Inflammatory Bowel Disease. ACS Nano.

[32] Li X., Fang S., Yu Y., Yang H., Rao Y., Hong D., Lu C., Yu M., Lu X., Yu C. (2022). Oral administration of inflammatory microenvironment-responsive carrier-free infliximab nanocomplex for the targeted treatment of inflammatory bowel disease. Chem. Eng. J..

[33] Kannan P.R., Chen L., Lv Y., Zhao R., Hu Y., Iqbal M.Z., Han Q., Kong X., Li Y. (2025). Smart Silk-Based In Situ Sol-Gel Modulates Rectal Microenvironment for Effective Ulcerative Colitis Alleviation. Adv. Heal. Mater..

[34] Gao C., Zhao W., Feng R., Zhang L., Ge L., Sun J., Zhang R. (2025). Melanin nanoparticles-loaded lactobacillus fermentum exosomes for targeted and visualized treatment of ulcerative colitis. J. Adv. Res..

