# Supplementary material for: Hydrogel-Shielded Ellagic Acid Nanoparticles Prolong Colonic Retention and Mitigate DSS-Induced Colitis via Reactive Oxygen Species Scavenging
Source: Foods. 2025 Jul 22;14(15):2559. doi: 10.3390/foods14152559 (PMC12346856; doi:10.3390/foods14152559)
Supplement: Supplementary file 1 [file foods-14-02559-s001.zip › foods-3735765-supplementary.pdf]

Supplementary materials

Table S1. Statistical parameters of prepared EAs.

Sample		PDI	Size	Figure
PEG1000	HRP3 mg	0.367	336.4	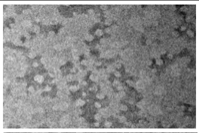
	HRP5 mg	0.14	239.4	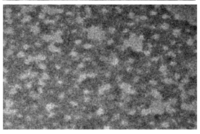
PEG2000	HRP3 mg	0.542	232	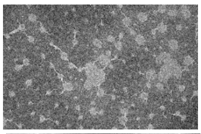
	HRP5 mg	0.393	368.4	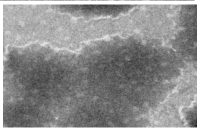
PEG4000	HRP3 mg	0.24	304.9	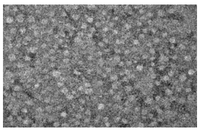
	HRP5 mg	0.1	26.59	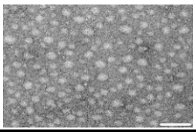

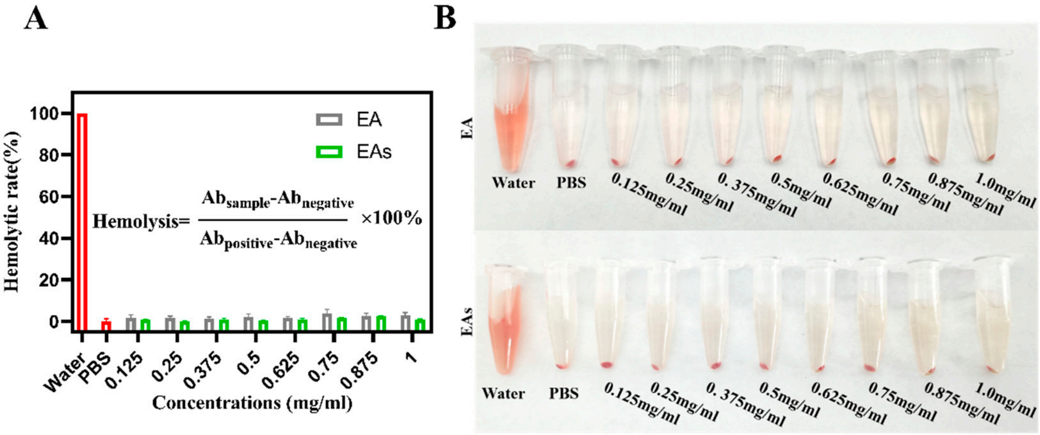

Figure S1. (A) Statistic analysis of the hemolysis rate (n=5); (B) Image of the result of hemolysis test.
